# Targeting mitochondrial structure and dynamics for therapeutic intervention in cancer

**DOI:** 10.1371/journal.pbio.3003453

**Published:** 2025-10-22

**Authors:** Wakiko Iwata, Nora Haggerty, Hiromi Sesaki, Miho Iijima

**Affiliations:** Department of Cell Biology, Johns Hopkins University School of Medicine, Baltimore, Maryland, United States of America

## Abstract

Mitochondrial division and fusion are critical regulators of cancer cell metabolism, proliferation, survival, metastasis, and drug resistance. Division promotes tumor development by reprogramming energy metabolism, whereas its inhibition can suppress tumor growth and metastasis. The mechanochemical GTPase DRP1, a key mediator of mitochondrial division, has emerged as a promising therapeutic target. Mitochondrial cristae also contribute to cancer progression by modulating metabolic reprogramming and oncogenic signaling. Targeting these processes may stimulate anti-tumor innate immune responses through the release of mitochondrial DNA into the cytoplasm. A deeper understanding of tumor-specific mitochondrial membrane structures and dynamics could therefore reveal novel intervention strategies and guide precision cancer therapies.

## Abstract 2

Mitochondrial dynamics are important regulators of various cancer cell phenotypes, including proliferation and metabolism. This Essay discusses the role of mitochondrial dynamic regulators in tumor growth and metastasis and explores how they can be targeted to develop novel intervention strategies.

## Introduction

Mitochondria are double-membrane-bound organelles often referred to as the powerhouses of the cell due to their central role in ATP production via oxidative phosphorylation. However, their functions extend far beyond energy generation, as they are also essential for biosynthesis, calcium homeostasis, redox balance, innate immune signaling, and apoptosis [1,2]. These diverse roles are supported by their unique structural features and adaptability. Unlike other organelles, mitochondria possess their own genome (mitochondrial DNA [mtDNA]) and a highly folded inner membrane that forms cristae, specialized structures that house the electron transport chain.

To meet changing cellular demands, mitochondria continuously remodel their architecture through fusion and division of the outer and inner membranes, as well as reorganization of cristae [3–5]. These dynamic processes allow cells to regulate mitochondrial morphology, distribution, and quality. Dysregulation of mitochondrial structure and dynamics contributes to numerous diseases, including neurodegeneration, metabolic disorders, and cancer. In tumors, altered mitochondrial architecture promotes metabolic reprogramming, stress resistance and enhanced survival, proliferation, and invasion [6–8]. In addition, disruption of mitochondrial dynamics or cristae integrity can lead to the release of mtDNA into the cytosol, thereby triggering innate immune responses through activation of pathways such as cGAS–STING (Box 1).

**Box 1. Immune responses triggered by altered mitochondrial dynamics and cristae.**

Aberrant mitochondrial division, fusion, and cristae remodeling can promote the release of mitochondrial DNA (mtDNA) from the matrix into the cytosol [9–11]. Studies have shown that the loss of mitochondrial proteins DRP1, MFN1, MFN2, OPA1, MIC60, MIC19, or SAM50 can lead to increased levels of cytosolic mtDNA in cultured cells and in mice [12–16]. In addition, simultaneous loss of the mitochondrial fusion regulators Parkin and OMA1 also results in mtDNA release in the mouse brain [17].

Although the precise mechanisms by which mtDNA escapes from the mitochondrial matrix remain largely unknown, two distinct outer membrane pore-forming mechanisms have been implicated. The first involves voltage-dependent anion channels, which can oligomerize to form large pores that permit mtDNA passage [18]. The second involves the pro-apoptotic proteins BAX and BAK, which co-oligomerize to form large channels in the outer mitochondrial membrane during apoptosis and senescence [19–21]. These findings suggest that normal mitochondrial dynamics and cristae architecture are essential for retaining mtDNA within mitochondria. Disruptions, whether structural or signaling-mediated, may lead to aberrant mtDNA release. Such release can either be a byproduct of mitochondrial dysfunction or a regulated component of the mitochondrial stress response that activates innate immune signaling.

Once in the cytosol, mtDNA activates cytosolic DNA-sensing pathways. In particular, mtDNA is a ligand for cyclic GMP–AMP synthase (cGAS), which binds double-stranded DNA in the cytosol. Upon binding DNA, cGAS synthesizes the second messenger cyclic GMP–AMP, which in turn binds to and activates the adaptor protein stimulator of interferon genes (STING) [9–11]. Activation of the cGAS–STING pathway triggers a cascade of innate immune responses, including the induction of interferon-stimulated genes and the production of pro-inflammatory cytokines and type I interferons. These interferons stimulate antigen-presenting cells, enhance tumor antigen presentation to T cells, and activate CD8 ⁺ cytotoxic T lymphocytes capable of eliminating tumor cells [22,23]. Additionally, natural killer cell activity is enhanced [24,25]. Therefore, mtDNA release acts as a pro-inflammatory trigger that can support anti-tumor immunity. Targeting mitochondrial dynamics such as division, fusion, and cristae remodeling may thus represent a novel anti-tumor strategy that modulates tumor signaling, metabolism, and immune responses.

In this Essay, we highlight key discoveries that have characterized the mechanisms of mitochondrial dynamics and structure and their roles in cancer development and progression, with an emphasis on tumor metabolism and metastasis. We also consider future developments and emerging strategies to harness these insights for novel therapeutic interventions.

## Mechanisms of mitochondrial dynamics

Mitochondria are highly dynamic and constantly undergo fusion and division, processes essential for maintaining their function, bioenergetic capacity, and cellular homeostasis [4,26,27]. Within the inner membrane, cristae form specialized architectures that are critical for oxidative phosphorylation and apoptotic signaling [4,28–30]. In cancer, aberrant mitochondrial dynamics such as excessive fragmentation and cristae remodeling have been implicated in cell proliferation, survival, and metastatic potential.

**Fig 1:**
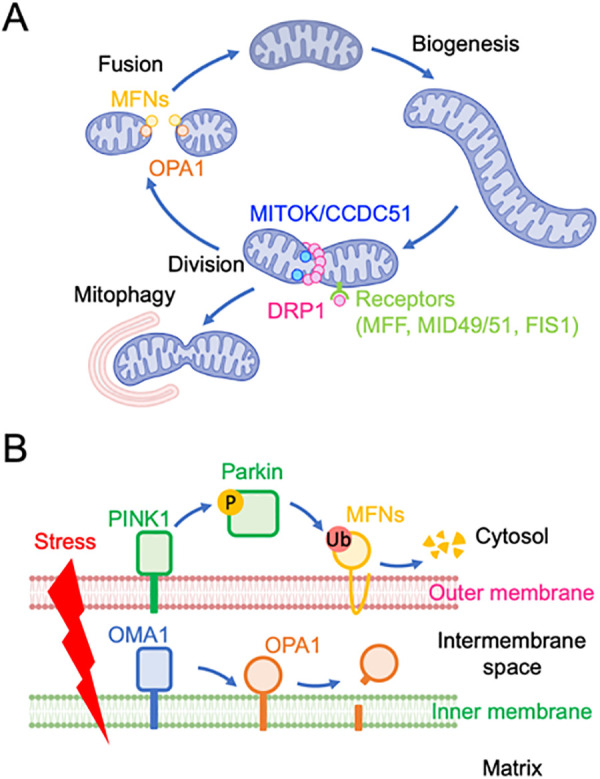
Mitochondrial division and fusion control mitochondrial structure. **A.** Mitochondrial division, mediated by DRP1, decreases mitochondrial size and increases their number. Mitochondrial fusion, controlled by MFNs and OPA1, counteracts division to maintain mitochondrial integrity. Mitochondrial degradation by mitophagy is influenced by mitochondrial size. **B.** Parkin and OMA1 negatively regulate mitochondrial fusion. Parkin is activated by phosphorylation mediated by the kinase PINK1. Activated Parkin ubiquitinates MFN1, leading to its degradation. OMA1 regulates mitochondrial fusion by cleaving OPA1.DRP1, dynamin-related protein 1; FIS1, fission protein 1; MFF, mitochondrial fission factor; MFN, mitofusin; MID49/51, mitochondrial dynamics proteins of 49 and 51 kDa; MITOK/CCDC51, mitochondrial ATP-sensitive potassium channel/coiled-coil domain-containing protein 51; OMA1, OMA1 zinc metallopeptidase; OPA1, optic atrophy 1; PINK1, PTEN induced kinase 1. *Created in BioRender. Sesaki,* H. *(2025)* *https://BioRender.com/kz9zz80*.

The balance between fusion and division governs mitochondrial morphology (Fig 1A). Excessive division produces smaller, fragmented mitochondria, whereas increased fusion results in more elongated, interconnected networks. Cells sense changes in mitochondrial size and adjust this balance accordingly; for example, when mitochondria become too large, fusion is actively downregulated through proteolytic processing of fusion proteins [17,31,32]. In this section, we summarize the core components of the mitochondrial division and fusion machinery and their regulation.

### Mitochondrial division

Mitochondrial division has a critical role in multiple aspects of mitochondrial and cellular physiology (Fig 1A) [5,7,27]. It allows mitochondria to increase in number during cell proliferation, contributes to proper mitochondrial distribution (especially in highly polarized cells such as neurons) and helps regulate mitochondrial size after organelles grow by importing proteins and lipids. Furthermore, the division is an essential component of mitochondrial quality control: it enables the segregation of damaged or dysfunctional mitochondrial segments, which can then be selectively removed via mitophagy [8,33,34]. This fragmentation is particularly important under conditions of stress, metabolic imbalance, aging, and tumorigenesis [35].

Mitochondrial division is primarily orchestrated by the cytosolic GTPase dynamin-related protein 1 (DRP1), which is recruited to the outer mitochondrial membrane by a set of mitochondrial-anchored receptor proteins, including mitochondrial fission factor (MFF), mitochondrial dynamics proteins of 49 and 51 kDa (MID49 and MID51), and fission protein 1 (FIS1) (Fig 1A). Studies have shown that different receptor proteins regulate distinct aspects of mitochondrial division [34,36,37]. MFF primarily mediates homeostatic division, maintaining organelle size and number under normal physiological conditions [38]. The function of MFF is regulated by phosphorylation through protein kinases such as AMPK and AKT [39,40]. By contrast, FIS1 facilitates quality control of division, associated with mitochondrial damage and subsequent clearance via autophagy [38]. MID49 and MID51 have more dynamic regulatory roles by linking mitochondrial division to cellular metabolism. These proteins bind fatty acyl-coenzyme A, acting as sensors of β-oxidation metabolites, thus coupling mitochondrial fission to changes in the cellular metabolic state [41,42].

In addition to outer membrane-anchored receptor proteins and cytosolic DRP1, recent studies have identified a role for intermembrane space proteins in mitochondrial division, including Atg44/Mdi1 and Mdm33 in yeast [43–47]. Atg44/Mdi1 seems to be yeast-specific [45], whereas Mdm33 is evolutionarily conserved and has a mammalian homolog known as mitochondrial ATP-sensitive potassium channel (MITOK, also referred to as CCDC51) [43,44]. MITOK/CCDC51 was initially identified as a pore-forming subunit of the mitochondrial potassium channel [48]. More recently, it was found to be required for the efficient completion of DRP1-mediated division [43]. Currently, these two roles of MITOK/CCDC51 are not clearly distinguished. It remains to be further investigated whether its involvement in mitochondrial division is mediated through potassium regulation or through an independent mechanism. While the role of DRP1 in cancer has been extensively studied (described in the following section), particularly concerning metabolic reprogramming and tumor cell migration, the function of newly identified components such as MITOK/CCDC51 remains largely unknown. Elucidating how inner membrane remodeling proteins such as MITOK/CCDC51 interface with the established fission machinery could uncover new regulatory mechanisms and therapeutic opportunities in diseases involving dysregulated mitochondrial dynamics.

### Mitochondrial fusion

Mitochondrial fusion is mediated by other dynamin-related GTPases, including mitofusin 1 (MFN1), mitofusin 2 (MFN2), and optic atrophy 1 (OPA1) (Fig 1A) [4,8,26]. MFN1 and MFN2 mediate fusion of the mitochondrial outer membrane, whereas OPA1 facilitates inner membrane fusion [29,49]. Efficient mitochondrial fusion requires the coordinated activity of both MFNs and OPA1; disruption of either results in impaired fusion and mitochondrial fragmentation.

Fusion is important for maintaining mitochondrial homeostasis by allowing mitochondrial complementation, whereby damaged mitochondria merge with healthier counterparts to dilute defective components and restore bioenergetic function [49,50]. This process is likely essential for limiting the accumulation of reactive oxygen species and for supporting efficient oxidative phosphorylation and other metabolic pathways. Particularly, mitochondrial fusion is crucial for optimal mitochondrial function and stress resilience in cells with high energy demands, such as neurons, muscle cells, and hepatocytes [51–53].

The fusion machinery is subject to tight regulation to ensure mitochondrial quality. For instance, the MFNs can undergo ubiquitin-mediated degradation, especially in response to mitochondrial damage, to prevent the fusion of dysfunctional organelles [32,33,54]. OPA1 is regulated through proteolytic processing by the inner membrane proteases OMA1 and YME1L, which modulate its activity in response to changes in membrane potential or other stress signals [17,55]. Loss of membrane potential activates OMA1, leading to cleavage of long (fusion-competent) OPA1 into short forms that are fusion-incompetent (Fig 1B). In contrast to DRP1, which is regulated by both activating and inhibitory signals, the known regulatory mechanisms of fusion GTPases primarily involve their suppression through proteolytic cleavage and degradation [17,55,56]. The mechanisms underlying their activation remain relatively poorly understood.

A recent study shows that the E3 ubiquitin ligase Parkin—which is defective in both familial and sporadic forms of Parkinson’s disease—synergistically regulates mitochondrial fusion with OMA1 under both physiological and stress conditions [38] (Fig 1B). Parkin promotes the ubiquitination and degradation of MFN1, thereby inhibiting outer membrane fusion, while OMA1 cleaves and inactivates OPA1, suppressing inner membrane fusion. Simultaneous loss of Parkin and OMA1 leads to the formation of giant mitochondria and leakage of mDNA [38,39]. Thus, the Parkin and OMA1 pathways act together to prevent excessive fusion at both the outer and inner mitochondrial membranes, preserving mitochondrial structure and maintaining cellular homeostasis [38].

## Mechanisms of cristae formation

**Fig 2:**
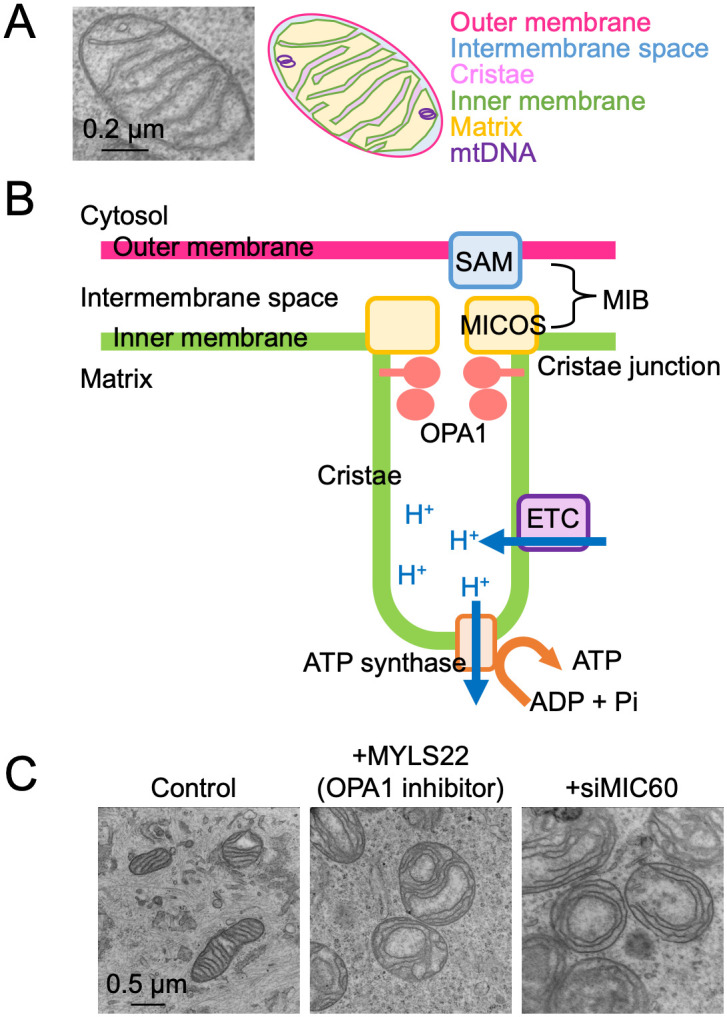
Structure and organization of cristae. **A**. Mitochondria possess two membranes: an outer membrane and an inner membrane. **B**. The inner membrane is organized into cristae by the coordinated action of MICOS, SAM, OPA1, and Complex V. MICOS and SAM interact to form the bridging supercomplex known as MIB. **C**. Inhibition of OPA1 by its specific inhibitor MYLS22 or knockdown of MIC60 by small interfering RNA disrupts normal cristae morphology in PANC-1 pancreatic carcinoma cells [67]. ETC, electron transport chain; MIB, mitochondrial intermembrane space bridging; MICOS, mitochondrial contact site and cristae organizing system; OPA1, optic atrophy 1; SAM, sorting and assembly machinery.

Cristae are invaginations of the inner mitochondrial membrane that serve to maximize membrane surface area for ATP production (Fig 2). These structures are molecularly distinct from the inner boundary membrane (the portion of the inner membrane that runs parallel to the outer mitochondrial membrane) [57–59]. The two regions are connected by narrow openings known as cristae junctions, which regulate the exchange of metabolites, ions, and proteins between the cristae lumen and the intermembrane space. Cristae folds form specialized subcompartments that optimize energy conversion, facilitate the assembly of electron transport chain supercomplexes, and support metabolic signaling [60,61]. In addition to its role in bioenergetics, seminal studies have demonstrated that cristae architecture is critical for apoptosis. Remodeling of cristae during the early stages of programmed cell death facilitates the release of cytochrome c into the cytosol, thereby initiating the apoptotic cascade [62–66].

Several key protein complexes govern the structure of cristae (Fig 2B). The mitochondrial contact site and cristae organizing system (MICOS) complex is a central organizer of cristae junctions and overall inner membrane architecture [68,69]. MICOS is a large, multi-subunit complex composed of MIC10, MIC13, MIC14, MIC19, MIC23, MIC25, MIC27, and MIC60.

The loss of MICOS alters cristae morphology but does not result in their complete absence (Fig 2C) [67]. It localizes to cristae junctions and promotes membrane curvature and stability [68,70]. MICOS also interacts with the sorting and assembly machinery (SAM) complex in the outer membrane to form a supercomplex known as the mitochondrial intermembrane space bridging (MIB) complex [68,70]. The MIB complex physically connects the inner and outer membranes, providing structural anchorage at cristae junctions. Interestingly, despite severe disorganization of the inner membrane structure, knockout of MICOS subunits in cultured HeLa cells only partially impairs mitochondrial respiration and the integrity of electron transport chain complexes [57].

In parallel, likely through coordination with the MICOS complex, the dynamin-related GTPase OPA1 also regulates cristae structure through oligomerization at the inner membrane [4,28,30]. OPA1 helps maintain cristae junctions, and its cleavage by stress-activated proteases such as OMA1 leads to cristae remodeling and facilitates cytochrome c release during apoptosis. The mechanisms by which OPA1 fuses the inner membrane and forms cristae are distinct. For example, while its GTPase activity is necessary for fusion, it is dispensable for cristae formation [71]. OPA1 functions upstream of MICOS in controlling the organization of cristae junctions [72]. Inhibition of OPA1 alters cristae morphology, similar to Mic60 depletion (Fig 2C).

Furthermore, at the extremely curved tips of tubular or lamellar cristae, the F_1_F_0_-ATP synthase (Complex V) not only generates ATP but also has a structural role (Fig 2B) [4,29,73].

Through oligomerization, ATP synthase bends the inner membrane by inducing positive curvature and helps stabilize the distinctive architecture of cristae. Notably, the assembly of Complex V requires OPA1, suggesting that ATP synthase acts as a key effector of OPA1 in controlling cristae morphology [71,74]. Detailed molecular mechanisms of cristae organization and the relationships among MICOS, OPA1, and F₁F₀-ATP synthase are comprehensively described in recent excellent reviews, which we recommend for a deeper understanding [30,49].

## Roles of mitochondrial dynamics and cristae in cancer

**Fig 3:**
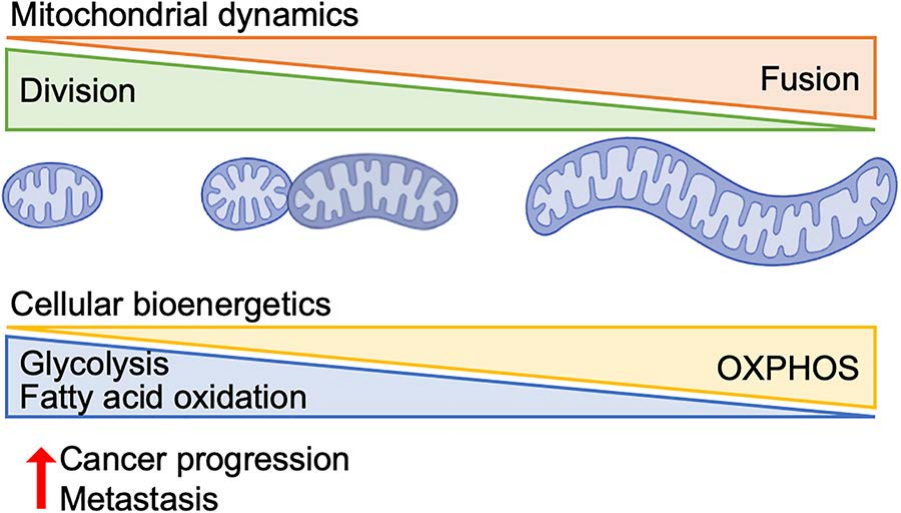
Mitochondrial dynamics influence cancer progression. During tumorigenesis, cancer cells promote mitochondrial fragmentation through DRP1-mediated division. Small, fragmented mitochondria enhance cancer progression, metastasis, and metabolic remodeling toward glycolysis and fatty acid oxidation. OXPHOS, oxidative phosphorylation. *Created in BioRender. Sesaki,* H. *(2025)* *https://BioRender.com/kz9zz80*.

Mitochondrial structure and function are shaped by dynamic remodeling processes, including division, fusion, and cristae organization, that enable cells to adapt to metabolic and environmental demands. In cancer, these processes are frequently hijacked to promote malignant transformation, metabolic reprogramming, and resistance to therapies. In this section, we highlight how alterations in each of these mitochondrial remodeling pathways contribute to tumor progression (Fig 3).

### Mitochondrial division and metastatic adaptation

Metabolic reprogramming is a hallmark of cancer cells, enabling them to meet the elevated demands for energy production, biosynthesis, and redox homeostasis necessary for rapid proliferation and survival [75,76]. Unlike normal cells, which primarily depend on mitochondrial oxidative phosphorylation, cancer cells often shift toward aerobic glycolysis: a phenomenon known as the Warburg effect (Fig 3) [77,78]. This metabolic shift allows for rapid ATP generation while simultaneously producing intermediates essential for the synthesis of nucleotides, amino acids, and lipids. In addition to enhanced glycolysis, many cancer cells reprogram glutamine metabolism, increase lipid biosynthesis, and modify mitochondrial function to support their growth and resist apoptosis. These metabolic alterations are driven by oncogenic signaling, loss of tumor suppressors, and cues from the tumor microenvironment, collectively making metabolic pathways attractive targets for cancer therapy [79,80]. Notably, mitochondrial division has emerged as a critical process facilitating these metabolic changes.

Several studies have reported that mitochondrial morphology correlates with metastatic potential. Breast cancer cells with low metastatic capacity exhibit elongated mitochondria, whereas highly metastatic cells display fragmented mitochondrial networks [81–83]. Inhibition of mitochondrial division reduces metastasis in vivo. Metabolomic analyses revealed that suppressing division leads to reduced levels of tricarboxylic acid cycle metabolites and increased cell adhesion, suggesting that impaired energy metabolism combined with enhanced adhesion slows metastatic spread [81–83]. In addition, small mitochondria generated by mitochondrial division localize to the leading edge of migrating leukocytes [84]. This positioning may promote AKT signaling and actin polymerization to drive cell migration by locally supplying the ATP required for these processes [84].

Latent brain metastases originating from breast cancer cells display elevated expression of genes involved in fatty acid metabolism, a metabolic pathway likely advantageous in the lipid-rich environment of the brain [85]. In these cells, mitochondria undergo fragmentation through DRP1-mediated mitochondrial division. Mechanistically, latent cells promote mitochondrial division by increasing DRP1 protein levels and its stimulatory phosphorylation [85]. This mitochondrial fragmentation enhances fatty acid oxidation, supporting bioenergetic demands and redox homeostasis. Inhibition of DRP1 impairs fatty acid oxidation, leads to intracellular lipid accumulation and significantly reduces the metastatic potential of these cells [85].

Similar to findings in breast cancer, in colon cancer cells DRP1 activation promotes both fatty acid oxidation and Wnt signaling [86]. Conversely, silencing DRP1 reduces mitochondrial respiration without affecting glycolytic flux [87]. Instead, glucose is diverted toward glycogenesis, resulting in intracellular glycogen accumulation. Combined inhibition of DRP1 and glycogenesis markedly suppresses colon cancer cell viability.

DRP1 also links mitochondrial division to oncogenic RAS signaling. During cellular transformation driven by oncogenic KRAS, DRP1 is phosphorylated by ERK1/2, promoting mitochondrial division and suppressing mitochondrial respiration [88,89]. In KRAS-driven pancreatic cancer, DRP1-mediated mitochondrial fragmentation enhances glycolytic flux by sustaining the activity of hexokinase 2 [90]. Hexokinase 2 catalyzes the first step of glycolysis, converting glucose into glucose-6-phosphate using ATP. Consistent with increased glycolytic activity, hexokinase 2 is highly expressed in many cancers and has a central role in the Warburg effect. Notably, genetic deletion of DRP1 suppresses pancreatic tumor development in vivo [90]. Furthermore, because DRP1 phosphorylation status governs cancer cell survival following inhibition of oncogenic MAPK signaling, targeting DRP1 and its phosphorylation may provide a pharmacological strategy to selectively eliminate cancer cells [89].

Recently, phospho-defective and phospho-mimetic DRP1 mice have been developed using CRISPR for the stimulatory phosphorylation site [91]. DRP1 was highly phosphorylated in the brain, and both mouse lines were viable and developed normally under physiological conditions, although they exhibited altered anxiety-like behaviors [91]. These models may serve as valuable tools to investigate the role of DRP1 phosphorylation in tumor development and metastasis in vivo.

From a therapeutic perspective, inhibiting mitochondrial division by targeting DRP1 was shown to effectively suppress chemoresistance across multiple cancer types, as mitochondrial elongation resulting from suppressed division enhanced drug-induced cell death [92]. This effect appears to be mediated by partial leakage of cytochrome c from mitochondria and an increased basal level of apoptosis. However, this finding contrasts with earlier work showing that loss of DRP1 renders cells resistant to apoptosis by suppressing cytochrome c release [93,94]. Therefore, DRP1 inhibition may provide an effective therapeutic approach when used in combination with anti-cancer drugs.

### Mitochondrial fusion and migration control

Mitochondrial fusion also has a multifaceted role in cancer, influencing not only mitochondrial morphology but also cell migration, metabolism, angiogenesis, and tumor growth [95,96]. Disruption of mitochondrial fusion can profoundly alter cancer cell phenotypes. Because overall mitochondrial morphology is determined by the dynamic balance between mitochondrial division and fusion, fragmentation can result from impaired fusion, while elongation can occur through increased fusion. Thus, mitochondrial fusion is potentially a critical regulator of diverse cancer-associated phenotypes.

As described above [85], DRP1 expression and phosphorylation are increased in invasive breast carcinoma and lymph node metastases, with mitochondria appearing more fragmented in metastatic breast cancer cells compared with non-metastatic breast cancer cells [83]. Knockdown of DRP1 or overexpression of the outer membrane fusion protein MFN1 significantly reduced invasive cell migration. Conversely, knockdown of MFN1 or MFN2 led to mitochondrial fragmentation and increased migration of breast cancer cells. The authors proposed that mitochondrial fragmentation promotes the redistribution of mitochondria to lamellipodia (the leading edge of migrating cells), where they locally generate ATP to fuel actin remodeling and motility [83]. By contrast, elongated mitochondria are excluded from lamellipodia, possibly due to their size, and suppress directional migration.

A critical regulator of MFN1 and MFN2 is the ubiquitin E3 ligase Parkin [97–99], which acts as a tumor suppressor in several cancer types [100–102]. Parkin expression reduces focal adhesion and suppress migration in multiple cancer cell types, including prostate cancer, independently of its role in mitophagy [103]. In a mouse model of prostate cancer, Parkin expression reduced both primary tumor growth and metastasis. However, interestingly, Parkin overexpression also promotes migration under certain conditions, suggesting context-dependent functions. Proteomic and ubiquitinomic analyses revealed a broad network of Parkin-regulated pathways associated with cell death, mitochondrial dynamics (both fusion and division), mitochondrial and endoplasmic reticulum proteostasis and pentose phosphate metabolism. Functionally, Parkin expression suppresses both mitochondrial fusion and division, possibly contributing to reduced mitochondrial remodeling and impaired motility in cancer cells [103].

### Cristae remodeling and tumor metabolism

Cristae remodeling is a key structural determinant of mitochondrial respiratory capacity and apoptosis regulation. The inner membrane GTPase OPA1 and the MICOS complex (e.g., MIC60) orchestrate cristae organization and integrity.

OPA1 has emerged as a key player in cancer biology. Unlike MFN1 and MFN2, this dynamin-related GTPase functions in both mitochondrial fusion and cristae organization. In breast cancer, high OPA1 expression correlates with poor prognosis [104]. Genetic or pharmacological inhibition of OPA1 reduces cell migration, proliferation, colony formation, and spheroid invasion in vitro and suppresses tumor growth in vivo [104]. Notably, silencing MFN1 or MFN2 did not impact these parameters, highlighting the distinct role of OPA1, likely through its regulation of cristae architecture rather than mitochondrial fusion. Loss of OPA1 did not impair mitochondrial respiration but led to the upregulation of tumor-suppressive microRNAs, including members of the miR-148/152 family, suggesting a transcriptional feedback mechanism linked to cristae remodeling.

In KRAS-driven pancreatic cancer, combined gene expression profiling analyses and patient survival data for the entire mitochondrial proteome converged on OPA1 as a critical regulatory node [67]. The pharmacological inhibition of OPA1 suppresses cell proliferation, spheroid growth, and migration and impairs mitochondrial respiration in pancreatic cancer cells. While knockdown of MFN1 caused mitochondrial fragmentation, it did not impair cell proliferation, reinforcing the conclusion that OPA1’s role in cristae organization, not fusion, is key to pancreatic cancer phenotypes. Supporting this, inhibition of MIC60, a core subunit of the MICOS complex responsible for cristae formation, also suppressed KRAS-driven cancer cell growth. Interestingly, OPA1 inhibition activated downstream KRAS signaling pathways, including AKT and ERK, potentially reflecting a compensatory response to mitochondrial stress. Combined inhibition of OPA1 and KRAS produced a synergistic effect, highlighting the therapeutic potential of dual targeting of mitochondrial cristae structure and oncogenic signaling [67].

In KRAS-driven lung adenocarcinoma, OPA1 is essential for tumorigenesis. OPA1 deletion reduces colony formation of KRAS-transformed cells in vitro, an effect rescued by DRP1 knockout, indicating a functional interplay between inner and outer membrane remodeling [105]. By contrast, in vivo, OPA1 loss inhibits KRAS-driven tumor development, but DRP1 loss fails to rescue the OPA1-null phenotype. However, deletion of MFN1 or MFN2 does not impair colony formation, further suggesting that OPA1’s role in cristae organization, rather than fusion, may be critical for supporting tumor growth.

OPA1 is also implicated in angiogenesis through a fusion-independent mechanism. NF-κB signaling drives angiogenesis by inducing pro-angiogenic factors such as vascular endothelial growth factor, interleukin-8, and matrix metalloproteinases. OPA1 is upregulated in endothelial cells in response to angiogenic stimuli and regulates cytosolic calcium levels, NF-κB activation, and angiogenic gene expression [106]. Endothelial-specific OPA1 deletion significantly inhibited tumor angiogenesis, growth and melanoma metastasis but did not impair mitochondrial respiration or ATP levels in mice [106]. Also, independent of its role in mitochondrial fusion, inhibition of OPA1, either by genetic depletion or by its specific inhibitor MYLS22 [107], selectively reduced mitochondrial respiration, proliferation, and migration in metastatic breast cancer cells [108].

The importance of OPA1 upregulation has been reported in therapy resistance in diverse cancers. In triple-negative breast cancer, residual tumors after chemotherapy show elevated OPA1 and oxidative phosphorylation, and sequential treatment with chemotherapy plus the OPA1 inhibitor MYLS22 prevents regrowth [109]. In acute myeloid leukemia, both genetic and pharmacologic inhibition of OPA1 disrupts cristae structure, reduces oxidative phosphorylation, and restores chemosensitivity, with efficacy demonstrated across multiple studies [110–112]. Further studies extended these findings to solid tumors, where OPA1 promotes mitochondrial integrity, metabolic fitness, and apoptotic resistance; OPA1 inhibitors such as MYLS22 and Opitor-0 synergize with BCL-2 and EGFR inhibitors to overcome drug resistance [107,108,113]. Collectively, these studies highlight its inhibition as a promising therapeutic strategy in both hematologic and solid malignancies.

A key regulator of OPA1 is OMA1, a stress-responsive metalloprotease in the inner mitochondrial membrane [32,114,115]. OMA1 activity increases under stress, leading to the cleavage of OPA1. In colorectal cancer cells, OMA1 is upregulated, and its expression correlates with poor patient survival [116]. Deletion of OMA1 suppresses colorectal cancer progression in xenograft mouse models [116]. In the hypoxic tumor microenvironment, OMA1 is further upregulated and cleaves OPA1, resulting in cristae disorganization and reduced levels of electron transport chain components [116]. This may shift the energy metabolism of colorectal cancer cells from oxidative phosphorylation to glycolysis, thereby promoting the Warburg effect. Furthermore, one study suggested that OMA1 may serve as a valuable prognostic marker and therapeutic target in osteosarcoma [117], whereas another showed that OMA1 knockdown reduces drug-induced cell death in T-cell acute lymphoblastic leukemia cells [118]. These contrasting findings indicate that the therapeutic benefit of targeting OMA1 is likely to be cancer type-specific, reflecting its multiple substrates and diverse roles in mitochondrial and cellular processes. Although targeting OMA1 may not be straightforward, an advantage is that its loss does not significantly impact animal health or physiology [17,119]. Thus, if certain cancer cells are highly dependent on OMA1, its inhibition could provide a therapeutic strategy with fewer adverse effects.

Whereas OMA1 modulates cristae remodeling through proteolysis of OPA1, the inner membrane protein MIC60 regulates cristae architecture through the MICOS complex. In prostate tumors, MIC60 expression is heterogeneous but often reduced [120]. MIC60 knockdown slows cell cycle progression while simultaneously decreasing apoptosis and promoting innate immune pathways and cytokine signaling, thereby enhancing epithelial-mesenchymal transition [120]. Additionally, MIC60 knockdown promotes spheroid invasion in vitro but reduces primary tumor growth in mice while enhancing metastatic potential [120]. These mixed phenotypes suggest that MIC60 contributes to multiple context-dependent processes in prostate cancer cells.

Building on the link between cristae structure and cancer progression, a recent study reported an intriguing regulation of mitochondrial metabolism associated with the presence or absence of cristae [121]. Cancer cells utilize various mitochondrial metabolic pathways, including oxidative phosphorylation and reductive proline biosynthesis. Individual cancer cells contain distinct types of mitochondria: oxidative and reductive. The proline biosynthetic enzyme P5CS assembles into filaments, reducing the number of cristae and promoting a reductive environment in a subset of mitochondria. By contrast, other mitochondria within the same cell maintain cristae and support oxidative metabolism [121]. These distinct mitochondrial environments are established by the presence or absence of cristae, regulated by P5CS, and maintained through mitochondrial fusion and division [121]. This regulation may support the complex energy metabolism requirements of cancer cells, which is heavily shifted toward glycolysis but still relies on mitochondrial oxidative phosphorylation for survival.

Together, these data highlight the importance of cristae integrity in sustaining tumor bioenergetics and stress resistance, and suggest that modulating inner membrane architecture may represent a novel therapeutic avenue in oncology.

## Future directions for therapy development

Among the targets discussed, DRP1 represents the most feasible and compelling therapeutic candidate. A large body of in vivo and in vitro evidence demonstrates that DRP1 activation correlates with poor prognosis, metastasis, and chemoresistance across multiple cancer types. Genetic deletion or pharmacological inhibition of DRP1 results in reduced tumor growth and metastasis. Moreover, its relatively discrete function as a cytosolic GTPase, activated by phosphorylation and recruited by specific receptors, makes it more amenable to targeted inhibition than inner membrane proteins embedded in complex architectures. Small molecule DRP1 inhibitors have shown promise in preclinical studies, but their specificity, bioavailability, and toxicity profiles remain a concern [122]. The development of selective DRP1 inhibitors with minimal off-target effects and favorable pharmacokinetics will be essential. In the future, next-generation agents could be engineered through structure-guided medicinal chemistry or by employing proteolysis-targeting chimeras to achieve degradation-based inhibition.

Furthermore, despite these promising attributes, the essential role of DRP1 in mitochondrial division, distribution, and quality control in normal cells necessitates careful evaluation of toxicity and the therapeutic window. Because mitochondrial dynamics are vital for tissues with high energy demands such as the heart, skeletal muscle, and neurons, systemic inhibition of DRP1 may impair normal physiology. One potential way to mitigate this risk is through tumor-specific delivery strategies, such as antibody–drug conjugates or nanoparticle-based systems, which could selectively target cancer cells, enhance tumor specificity, and reduce systemic toxicity, thereby improving therapeutic efficacy.

By contrast, targeting mitochondrial fusion proteins, particularly MFN1 and MFN2, may be less fruitful. Despite their clear roles in shaping mitochondrial morphology, their impact on cancer phenotypes is highly context-dependent and often modest. Unlike DRP1, MFN1/2 suppression does not consistently affect tumor growth or metabolism across models [104,105]. Furthermore, their redundant or compensatory functions complicate therapeutic targeting. Additionally, given their essential role in maintaining mitochondrial health in high-energy tissues such as the heart and brain, systemic inhibition of MFN1/2 could carry significant toxicity risks. Therefore, while MFNs are mechanistically interesting, their clinical utility as direct drug targets is limited.

OPA1 presents a more nuanced case. While OPA1 functions in both fusion and cristae organization, the latter appears to be the more critical determinant of its cancer-promoting activity. Studies in breast, lung, and pancreatic cancers highlight OPA1’s role in supporting tumor metabolism and survival, particularly via its influence on cristae integrity [105]. Inhibiting OPA1 disrupts tumor growth and sensitizes cells to stress. However, OPA1’s dual role complicates selective targeting. Nevertheless, the specificity of its function in cancer versus normal tissues — particularly its unique role in cristae maintenance—offers a rationale for further exploration. Targeting OPA1’s fusion-independent functions through allosteric modulators or conformationally selective inhibitors could become a promising area for rational drug design. From a feasibility standpoint, OPA1 is more accessible than MICOS components but less so than DRP1 due to its inner membrane localization, although delivery challenges could be addressed through mitochondrial-targeted formulations.

Similarly, the MICOS complex, particularly MIC60, has a central role in cristae architecture. However, the pleiotropic and sometimes paradoxical phenotypes observed upon MIC60 depletion (e.g., reduced primary tumor growth but increased metastasis) underscore the complexity of targeting cristae organizers. The heterogeneous expression of MIC60 in tumors and the incomplete understanding of how its dysfunction affects bioenergetics, immune signaling, and metastatic programs further complicate the therapeutic outlook. In addition, the multi-subunit and membrane-embedded nature of MICOS complexes limits their usefulness as drug targets. Future strategies may involve precision medicine approaches that exploit MIC60 levels or mutations as biomarkers for patient stratification. Synthetic lethality screens could also reveal cancer-specific vulnerabilities in MIC60-deficient tumors, creating new avenues for combination therapies. At present, MICOS targeting is best viewed as an emerging concept, rather than a viable near-term drug strategy.

OMA1, a stress-responsive protease that cleaves OPA1, stands out as a promising cancer-specific vulnerability. Upregulated in hypoxic tumor environments and dispensable in normal tissues, OMA1 deletion selectively impairs tumor progression and cristae remodeling without overt toxicity in animal models [116]. Given this specificity and the emerging evidence that OMA1 promotes the Warburg effect and suppresses mitochondrial integrity in tumors, it may represent a high-reward target for pharmacological inhibition. Still, the development of specific OMA1 inhibitors remains in its infancy. A promising direction would be to develop substrate-mimetic or activity-based covalent inhibitors that trap the protease in a nonproductive state.

In summary, mitochondrial division, particularly via DRP1, is broadly tumor-promoting and represents a strong, actionable target for therapy. Fusion components, especially MFNs, are less attractive due to redundancy and essential roles in noncancerous tissues. By contrast, OPA1, OMA1, and cristae regulators such as MIC60 are emerging as modulators of cancer metabolism and signaling, but their therapeutic targeting requires a deeper mechanistic understanding and context-specific validation. Importantly, combining mitochondrial dynamics modulation with inhibitors of oncogenic signaling (e.g., KRAS or ERK) or immunotherapy may enhance anti-tumor efficacy while overcoming resistance. As such, mitochondrial-targeting agents may serve as sensitizers or potentiators of current treatments, including immune checkpoint blockade and metabolic inhibitors. Furthermore, because mitochondrial structural changes resulting from an imbalance between fusion and division can promote mtDNA release and immune activation, modulating mitochondrial dynamics may induce a robust anti-tumor immune response (Box 1).

## Conclusions

Mitochondrial structure—regulated by division, fusion, and cristae formation—has emerged as a critical determinant of various cancer cell phenotypes, including proliferation, metabolism, signaling, and metastasis (Fig 3). The balance between fusion and division governs mitochondrial morphology and function, with excessive division leading to fragmentation that can fuel tumor progression by reprogramming energy metabolism and facilitating cellular adaptation to stress and migration. However, changes in mitochondrial dynamics alone do not drive tumor development or progression; these processes are primarily driven by mutations in oncogenes and tumor suppressor genes. Rather, alterations in mitochondrial dynamics enable cancer cells to remodel metabolism to adapt to hypoxic environments, enhance migration during metastasis and acquire resistance to cell death during chemotherapy. As our understanding of mitochondrial plasticity and its crosstalk with oncogenic and immune pathways grows, targeting mitochondrial dynamics is poised to become a frontier in next-generation cancer therapies.

## Abbreviations

cGAS: cyclic GMP–AMP synthase
DRP1: dynamin-related protein 1
FIS1: fission protein 1
MFF: mitochondrial fission factor
MFN1: mitofusin 1
MFN2: mitofusin 2
MIB: mitochondrial intermembrane space bridging
MICOS: mitochondrial contact site and cristae organizing system
MID49: dynamics proteins of 49 kDa
MID51: dynamics proteins of 51 kDa
MITOK/CCDC51: mitochondrial ATP-sensitive potassium channel
mtDNA: mitochondrial DNA
OPA1: optic atrophy 1
SAM: sorting and assembly machinery
STING: stimulator of interferon genes
